# A false-pouch closure technique with an intact superior peroneal retinaculum for recurrent dislocation of the peroneal tendon

**DOI:** 10.1186/s40634-021-00343-0

**Published:** 2021-03-18

**Authors:** Tomohiro Matsui, Tsukasa Kumai, Yasushi Shinohara, Noriyuki Kanzaki, Koji Noguchi, Hirofumi Tanaka, Takeshi Sugimoto, Hiroki Yabiku, Ichiro Higashiyama

**Affiliations:** 1 Department of Orthopaedic Surgery, Saiseikai Nara Hospital, Nara, Japan; 2 Faculty of Sports Sciences, Waseda University, 2-579-15 Mikajima, Tokorozawa city, Saitama Japan; 3 Faculty of Sport and Health Science, Ritsumeikan University, Kusatsu, Japan; 4 Department of Orthopaedic Surgery, Kobe University Graduate School of Medicine, Kobe, Japan; 5 Department of Orthopaedic Surgery, Kurume University Medical Center, Kurume, Japan; 6 Department of Orthopaedic Surgery, Hyakutake Orthopaedic & Sports Clinic, Saga, Japan; 7 Department of Orthopaedic Surgery, Maki Orthopaedic Hospital, Osaka, Japan; 8 Department of Orthopaedic Surgery, Matsukura Hospital, Nara, Japan

**Keywords:** Peroneal tendon, Dislocation, Reattachment, False pouch

## Abstract

**Purpose:**

To evaluate the usefulness of the false-pouch closure technique with an intact superior peroneal retinaculum (SPR).

**Methods:**

From 2016 to 2020, 30 patients with recurrent dislocation of the peroneal tendon were treated with the current procedure. Clinical outcomes, including the time to resume running, the rate and time to return to sports, and the American Orthopaedic Foot and Ankle Society (AOFAS) Ankle-Hind Foot score, were evaluated preoperatively and at the last follow-up.

**Results:**

The rate of return to the pre-injury level of sports activity was 93.3%, and the mean duration to return to running and sports was 8.0 ± 2.8 weeks (range: 3–12 weeks) and 14.4 ± 3.2 weeks (range: 10–24 weeks), respectively. The mean preoperative AOFAS score was 79.7 ± 9.6 points (range: 41–90), which improved significantly to 98.9 ± 3.2 (87–100) postoperatively (*p* < 0.01).

**Conclusion:**

The false-pouch closure technique with suture tape and anchors had a reliable clinical outcome and can enable the early return of patients to their sports activities.

**Level of evidence:**

IV, Case series

## Background

Dislocation of the peroneal tendon is as a result of injury to the superior peroneal retinaculum (SPR) or avulsion fracture at the attachment site of the SPR to the fibula; this injury is often related to sports activities. The failure rate of conservative treatment for acute dislocation of the peroneal tendon is reported to be approximately 50%. It is known to further result in recurrent dislocation. Previous studies have reported that in most patients with recurrent dislocation, false pouches made on the lateral malleolus often responded poorly to conservative treatment. Surgical treatment is required for symptomatic recurrent dislocation [14, 25]. Das De et al. reported an anatomical SPR reattachment procedure and preferable clinical outcomes [3]. Although an excellent clinical outcome was reported for the reattachment procedure, Cho et al. reported recurrence in one patient after SPR reattachment and mentioned that loose suturing of the SPR may cause recurrence [3]. It is important to improve the initial strength by fixing the SPR so that postoperative treatment can be accelerated, resulting in early return to sports activity. The purpose of this study was to report the technique of false-pouch closure with suture tape and anchors and its short term clinical outcomes.

## Methods

The study data were retrospectively collected from multicentre databases. A total of 30 patients were treated with the current procedure from 2016 to 2020 by six surgeons at these centres. There were 19 men and 11 women. The right foot was affected in 10 patients; and the left foot, in 20. The mean age was 22.0 ± 8.4 years (range: 14–42 years), and the mean follow-up duration was 10.8 ± 6.2 months (range: 2–33 months). The cause of all injuries was related to sports activities, as shown in Table 1. The inclusion criteria for this study were recurrent dislocation of the peroneal tendon without any bony fragment and torn retinaculum. The indication of the current procedure was type 1 of Oden’s classification (Table 2), in which the SPR is still attached to the periosteum of the lateral malleolus; however, the periosteum is elevated from the underlying malleolus and makes a false-pouch [19]. The cases in which the SPR avulsed from the insertion on the malleolus with an avulsion of a small fragment of bone and those in which the SPR was torn at insertion or mid-substance were excluded from the indication criteria for the current procedure.

**Table 1:** Patients’ characteristics

**Case**	**Age (y)**	**Sex**	**Affected side**	**Sports**	Follow-up (Months)
1	20	F	L	Volleyball	33
2	16	M	L	Soccer	6
3	31	M	L	Soccer	2
4	16	M	L	Basketball	12
5	17	M	L	Basketball	16
6	17	M	R	Soccer	18
7	16	F	L	Basketball	10
8	42	M	L	Karate	7
9	15	F	L	Basketball	7
10	20	F	L	Soccer	17
11	37	M	L	Triathlon	14
12	27	F	R	Skiing	8
13	38	M	R	Baseball	6
14	15	F	L	Tennis	18
15	22	M	L	Rugby	19
16	19	M	L	Rugby	12
17	20	M	R	Rugby	12
18	17	F	L	Badminton	6
19	19	M	L	Rugby	12
20	19	F	R	Gymnastics	7
21	21	M	R	Badminton	18
22	23	M	R	Rugby	12
23	19	M	R	Table tennis	9
24	15	F	R	Soccer	6
25	15	F	L	Soccer	6
26	14	F	L	Basketball	6
27	15	M	R	Baseball	6
28	41	M	L	Skiing	6
29	18	M	L	Baseball	6
30	36	M	L	Soccer	6

**Table 2:** Oden classifications

Type1	SPR is elevated from the lateral malleolus and FCR, but SPR is still attached to the periosteum of the fibula
Type2	SPR is torn free from its attachment to the lateral malleolus
Type3	A small fragment of the bone is avulsed at the attachment of the SPR
Type4	SPR is torn from its posterior attachment and is usually lying deep to the tendon

**Fig. 1:**
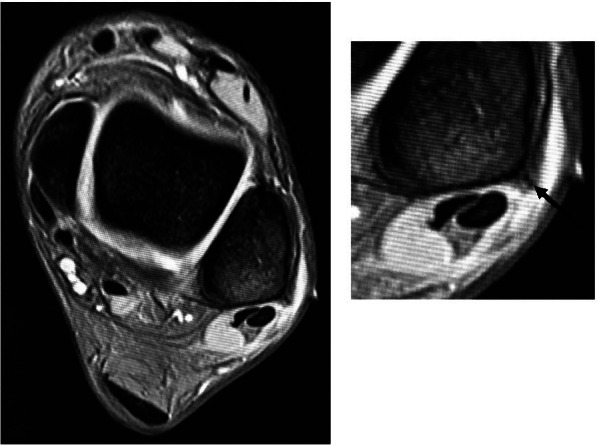
Preoperative MRI (T2WI). MRI showing a formed false-pouch, although the fibrocartilaginous ridge (arrow) remains intact

**Fig. 2:**
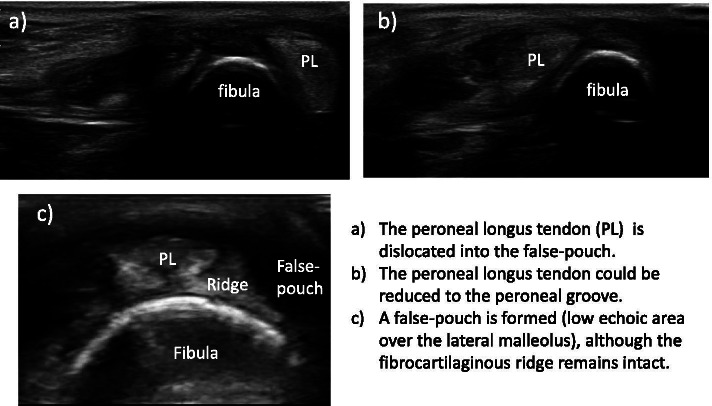
Preoperative US image

Imaging studies were performed for all the participants. Plain radiography and computed tomography (CT) revealed no bony fragments suggestive of avulsion fractures. Magnetic resonance imaging (MRI) revealed no tear in the peroneal longus and brevis tendons. The SPR and fibula periosteum were found detached from the fibula forming a false-pouch, while the fibrocartilaginous ridge remained intact (Fig. 1). Ultrasonographic (US) imaging also revealed the presence of false pouch and continuity of the SPR, and that the peroneal longus tendon could be dislocated from the bony groove (Fig. 2).

One patient experienced recurrent dislocation after undergoing a reattachment procedure twenty-eight years earlier.

### Operative technique

The operations were performed under regional anaesthesia. The patient was placed in the lateral decubitus position, and a pneumatic tourniquet was placed around the lower thigh.

**Fig. 3:**
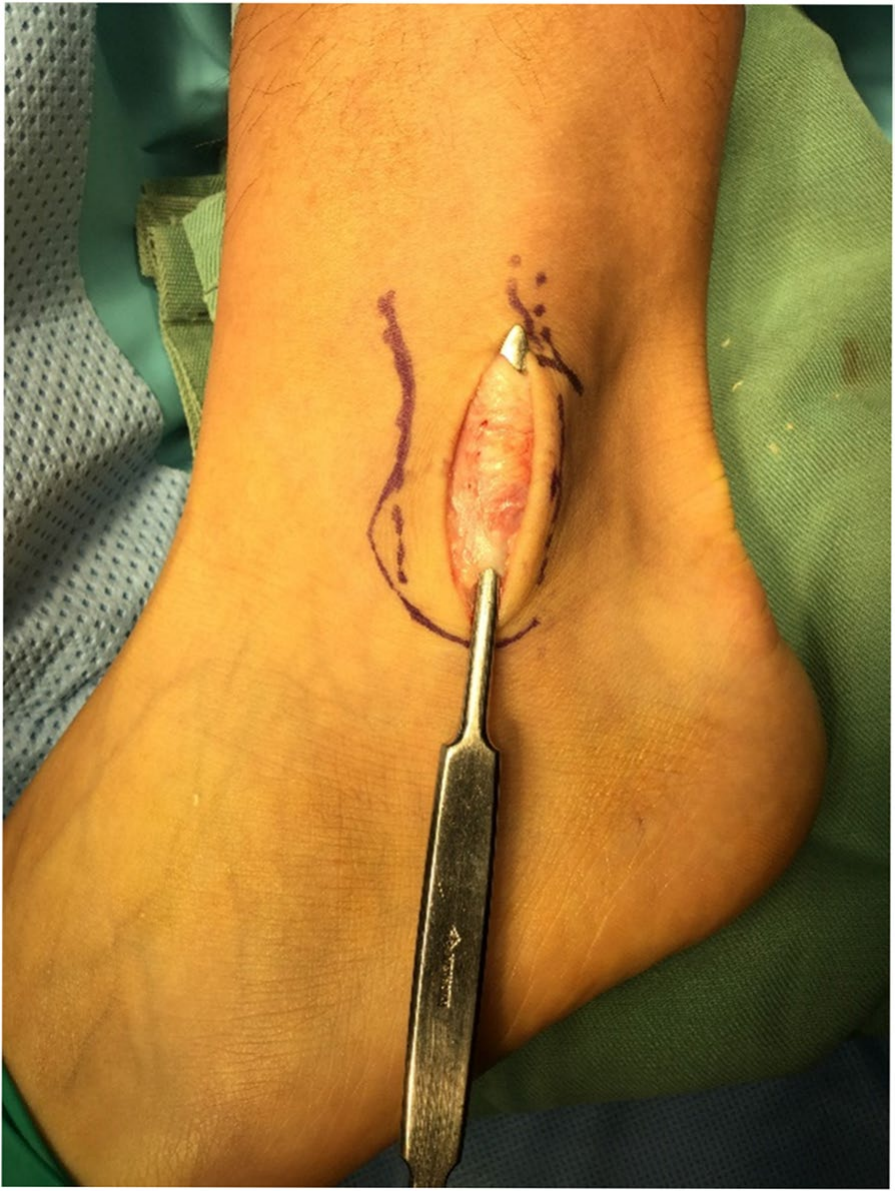
Opening of the proximal and distal ends of the false-pouch. Small incisions are made at the proximal and distal ends of the false-pouch, and the surface of the fibula is rasped

**Fig. 4:**
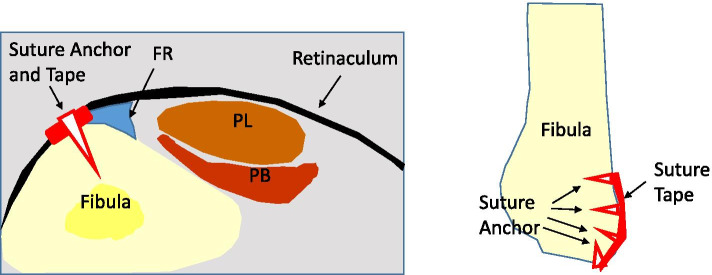
Schema of fixation with suture tape. The suture tape is fixed with 3 or 4 suture anchors. The suture anchors are inserted just anterior to the fibrocartilaginous ridge (FR). PL: Peroneus longus tendon. PB: Peroneus brevis tendon

**Fig. 5:**
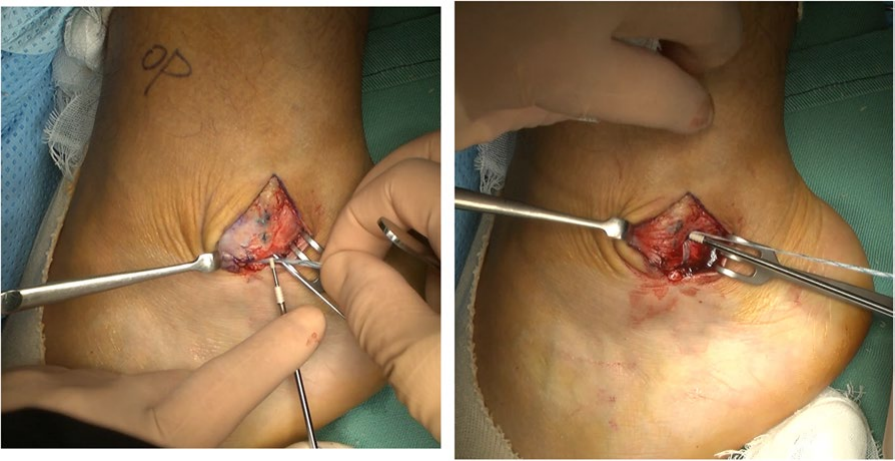
Direction of the anchors inserted. First, the anchor is inserted from the tip of the lateral malleolus in the direction of the fibula head. (Left) Other three suture anchors are inserted at an angle of 30° from the lateral aspect of the fibula. (Right)

**Fig. 6:**
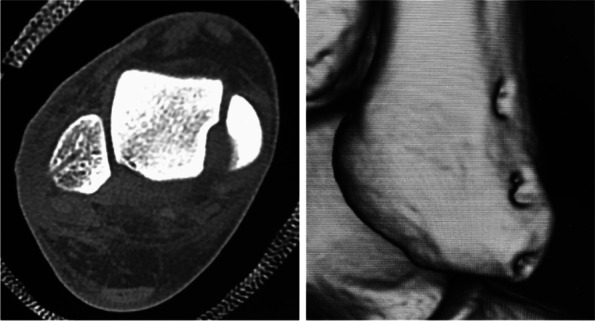
Postoperative CT image. Postoperative CT image showing the position and direction of the anchor holes

Marks were made on the skin at the proximal and distal ends of the false pouch as determined by US imaging before the start of the operation. A longitudinal incision from the tip of the fibula to the proximal end of the false pouch, usually 3–4 cm long, was made over the lateral aspect of the fibula. The superior peroneal retinaculum was exposed, with usually no tear detected; however, the retinaculum and periosteum of the fibula were elevated off the fibula and had made a false pouch. The peroneal tendon, which was dislocated, was manually displaced to the false pouch. Small incisions were made at the proximal and distal ends of the false pouch, and from there, rasp was put into the false pouch to refresh the surface of fibula so that bleeding from the soft tissue would promote adhesion of the repaired retinaculum and periosteum to the fibula (Fig. 3). Suture tape was placed over the periosteum just anterior to the fibrocartilaginous ridge and fixed with 3 or 4 suture anchors (Fig. 4). For the first 6 cases, 3 suture anchors (φ3.5-mm DEX Swive Lock™; Arthrex, Naples, FL) were used to fix the 2-mm wide suture tape (Arthrex, Naples, FL). A suture anchor (φ1.6-mm Fibre Tak™; Arthrex, Naples, FL) with a 1.3-mm width suture tape (Arthrex, Naples, FL) was added at the tip of the fibula, and the other 3 suture anchors were changed to smaller-sized suture anchors (φ2.5-mm mini Push Lock™; Arthrex, Naples, FL) for the next 24 cases. A Fibre Tak™ anchor was inserted from the tip of the lateral malleolus in the direction of the fibula head, and the other 3 suture anchors were inserted in the lateral aspect of the fibula just anterior to the fibrocartilaginous ridge at an angle of 30° against the lateral aspect of the fibula (Fig. 5) [9]. The suture tape was covered with subcutaneous tissue, and the skin was closed routinely. Postoperative CT imaging revealed anchor positions and directions (Fig. 6).

### Postoperative management

A short-leg cast in the neutral position of the ankle joint was applied for 2–3 weeks postoperatively. After cast removal, partial weight-bearing was permitted, and full weight-bearing was permitted at 2–4 weeks. Running was allowed after 4–6 weeks, and return to play at the pre-injury level was allowed after 10 weeks.

### Evaluation

The American Orthopaedic Foot and Ankle Society (AOFAS) Ankle-Hind Foot score was evaluated preoperatively and at the last follow-up for the patients who were followed up for > 6 months. The time to resume running and the rate and time to return to sports activity were recorded. The time to return to sports was defined as the time when the patient could return to the preoperative level of sports activity. The incidence rates of postoperative recurrent dislocation and other complications were also recorded.

### Statistical analysis

The AOFAS scores before operation and at the last follow-up were compared using the paired Student *t* test in Microsoft Excel 2013 (Microsoft Corp., Washington, USA). Significance was reported at the 95% confidence level (*p* < 0.05).

## Results

**Fig. 7:**
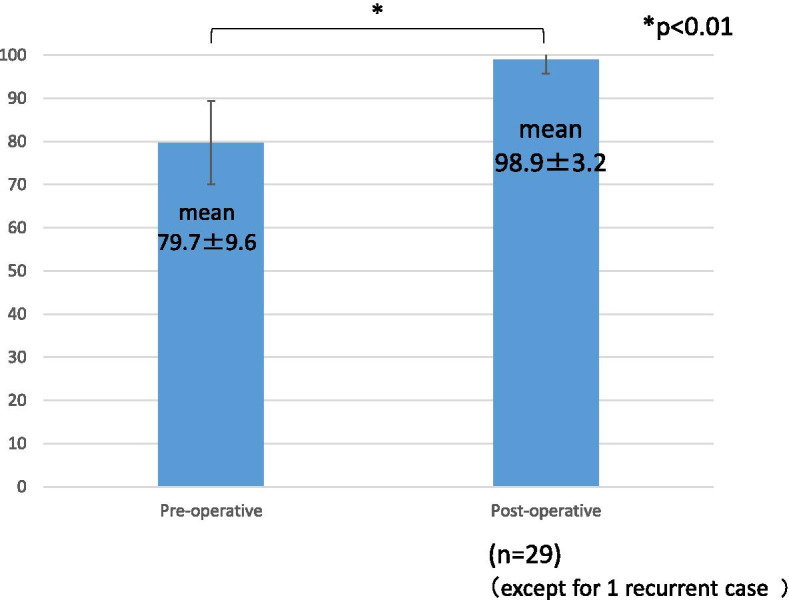
AOFAS Ankle-Hind Foot scale scores

One patient had a postoperative recurrent dislocation at 8 weeks from operation when joining a professional soccer team and trained to return to play, and underwent another operation for recurrent dislocation. Another patient did not return to the preoperative level of sports activity for reasons other than the ankle condition. The other 28 patients (93.3%) were able to return to their preoperative level of sports activity without any major complications. The mean time to resume running was 8.0 ± 2.8 weeks (range: 3–12 weeks) and time to return to sports was 14.4 ± 3.2 weeks (range: 10–24 weeks; Table 3). The AOFAS score was evaluated for 29 patients who did not undergo another operation for the peroneal tendon. The mean preoperative AOFAS score was 79.7 ± 9.6 (range: 41–90), which improved significantly to 98.9 ± 3.2 (87–100) postoperatively (*p* < 0.01; Fig. 7).

**Table 3:** Results

Case	Runnning (weeks)	Return to play (weeks)	Recurrence	Pre AOFAS scale score	Post AOFAS scale score
1	10	16	-	82	100
2	8	16	-	41	87
3	6	-	+	68	-
4	12	15	-	85	100
5	12	16	-	85	100
6	8	14	-	82	100
7	6	12	-	85	100
8	10	18	-	72	90
9	5	10	-	87	100
10	12	24	-	87	100
11	10	16	-	82	100
12	12	20	-	80	100
13	5	12	-	87	100
14	6	12	-	78	92
15	8	16	-	70	100
16	4	10	-	73	100
17	4	12	-	73	100
18	3	12	-	87	100
19	4	12	-	87	100
20	8	12	-	87	100
21	7	16	-	87	100
22	6	12	-	74	100
23	7	-	-	90	100
24	12	15	-	79	100
25	12	15	-	69	100
26	8	12	-	85	100
27	8	12	-	77	100
28	8	16	-	87	100
29	7	12	-	84	100
30	11	19	-	69	100

## Discussion

The most important finding of the present study was that the SPR reattachment procedure with suture tape was a safe and effective procedure for treating the dislocation of the peroneal tendon.

Many types of surgical procedures for dislocation of the peroneal tendon have been reported, such as reattachment of the SPR [1–3, 5, 6, 12, 18, 23, 24], bone block procedures [7, 10, 15, 24], groove deepening procedures [2, 8, 27], rerouting procedures [13, 21], and tissue transfer procedures [16, 17]. Among these procedures, reattachment of the SPR is known as an anatomical and less invasive procedure.

The Das De procedure (Singapore operation) is a popular reattachment procedure and has been reported to have good clinical outcomes [3, 6]. However, it is usually followed by 6 weeks of cast immobilization [3, 6], which is a major disadvantage of this procedure. Recently, the Das De procedure was modified by some surgeons and, thereafter, accelerated postoperative treatment [1, 2, 4, 5, 10, 12, 18, 23, 26]. The modified Das De procedure requires only one suture line on SPR to close the false-pouch and has the advantage of allowing repair while preserving the blood supply of the SPR compared to the original Das De procedure, which requires two suture lines [23, 24]. However, Cho et al. [2] and Deng et al. [5] mentioned that loose suturing of the SPR may cause postoperative recurrent dislocation.

There are some advantages of the current procedure to the traditional pouch closure technique. First, suture tape can be fixed to a wide area of the SPR. Further biomechanical studies are needed to confirm whether the current procedure can fix SPR more firmly to the bone than the previously reported false-pouch closure technique. Previous biomechanical studies have shown that a wider contact area with the suture bridge technique has superior time-zero structural properties in rotator cuff repair [20, 22].

Second, the current procedure does not require an incision to the SPR to improve healing through better preservation of the blood supply, and less peritendinous fibrosis will not cause tendon irritation and adherence after the operation. The endoscopic procedure also requires no incision to the SPR and is less invasive, though technically demanding and involves a steep learning curve [11, 18]. Current procedure has advantage in ts ease of learning compared to endoscopic procedure.

Third, the suture anchor is knotless; therefore, the current technique is advantageous in areas with poor subcutaneous tissue, such as the area around the lateral aspect of the fibula. Suture tape also has a lower profile; thus, there should be no hardware irritation.

Care must be taken when inserting the anchors in the lateral malleolus. The fibula is a thin bone; therefore, a short small-diameter anchor is needed, this was the reason why the authors changed the suture anchors for the present cases.

The limitations of the current study include the fact that the follow-up period was too short to obtain the necessary follow-up clinical results and data on dislocation recurrence. Biomechanical studies are needed to investigate the strength of the initial fixation, identify the number of suture anchors that should be used, and the interval between suture anchors. Further studies are also needed to determine the optimal protocol for postoperative treatment.

## Conclusion

A false-pouch closure technique with suture tape and anchors was described, which was found to have reliable results and to enable early return of patients to their sports activities.

## Abbreviations

SPR: Superior peroneal retinaculum
CT: Computed tomography
MRI: Magnetic resonance imaging
US: Ultrasonography
AOFAS: American Orthopaedic Foot and Ankle Society
